# Correlates of Patient Trust in Doctors: Demographic Factors and Experiences of Medical Care Discrimination

**DOI:** 10.1007/s11606-025-09474-x

**Published:** 2025-03-31

**Authors:** Lindsey M. Mundy, Suzanne E. Judd, Olivio J. Clay, Virginia J. Howard, Raegan W. Durant, Erin E. Ballard, Michael Crowe

**Affiliations:** 1 Department of Psychology, University of Alabama at Birmingham (UAB), Birmingham, AL USA; 2 Department of Biostatistics, School of Public Health, UAB, Birmingham, AL USA; 3 Alzheimer’s Disease Research Center, UAB, Birmingham, AL USA; 4 Department of Epidemiology, School of Public Health, UAB, Birmingham, AL USA; 5 Department of Medicine, Heersink School of Medicine, UAB, Birmingham, AL USA

**Keywords:** Physician-patient relationship, Race, Healthcare inequity

## Abstract

**Background:**

When providing healthcare services to diverse populations of middle-aged and older adults, it is important to understand factors that may influence the amount of trust they have in their doctors, such as demographic factors and previous experiences of discrimination.

**Objective:**

We examined correlates of general trust in doctors in a national sample of adults in the USA.

**Design:**

The REGARDS longitudinal cohort study included measures of trust in doctors and discrimination at a follow-up visit. Cross-sectional sequential linear regression models, with general trust in doctors as the outcome, first included demographic factors and then added discrimination in a medical care setting.

**Participants:**

The baseline REGARDS sample included community-dwelling participants across the contiguous USA who identified as White or Black/African American and were aged 45 or older. Our analytic sample included 8500 participants who completed the second in-home REGARDS visit and were aged 52 years or older.

**Main Measures:**

Trust was measured by the General Trust in Doctors Scale. Participants also reported whether they had ever experienced discrimination in a medical care setting.

**Key Results:**

Female sex (*b* = −1.41, *p* < 0.05), Black/African American race (*b* = −0.40, *p* < 0.05), and having a higher level of education (*b* = −0.45, *p* < 0.05) were each independently related to lower trust in doctors. Older age (*b* = 0.10, *p* < 0.05) was associated with higher trust. Previous discrimination had a negative association with trust (*b* = −4.27, *p* < 0.05) and the relationship between race and trust was reduced to zero (*b* = 0.28, *p* = 0.155) with discrimination in the model.

**Conclusions:**

Previous discrimination experiences in a medical care setting completely attenuated the relationship between race and trust in doctors, a prominent finding that should be considered when providing healthcare services to diverse populations of adults.

## INTRODUCTION

Trust is crucial in the relationship between patients and doctors. Lower levels of trust in doctors are linked to worse patient health outcomes,^1,2^ lower medication adherence,^3,4^ less willingness to disclose important information to the healthcare team,^5^ and missed opportunities for support and utilization of preventive healthcare.^6,7^ These outcomes are not only harmful for the individual, but also costly for the entire healthcare system.^8^ On the other hand, higher trust in healthcare professionals, such as doctors, is connected to higher treatment satisfaction, better quality of life, and positive health behaviors.^9^

Prior research has explored numerous predictors of trust in doctors, including age,^10^ race,^10–12^ gender or sex,^13^ education,^10,13–15^ and urban/rural population size.^16^ In addition, personally experiencing discrimination in a medical care setting has been found to influence trust in doctors.^17^ However, discrimination is not equally distributed; thus, certain groups may experience a greater impact. Being a Black/African American individual is strongly tied to experiences of discrimination in general^18^ and is associated with lower trust in doctors^11^ in clinical samples. However, the link between discrimination, race, and trust in doctors has not been studied in national community samples.

For instance, interpersonal experiences of discrimination (e.g., actions between individuals that negatively affect the person being discriminated against) while receiving healthcare were previously found to mediate the relationship between Black/African American race and lower physician trust in a sample of 430 older Black/African American and White veterans with osteoarthritis.^17^ In another study, discrimination was associated with decreased medication adherence in a sample of 780 Black/African American older adults with hypertension, and this association was mediated by physician trust.^19^ These findings highlight the exigency of understanding the relationship between experiencing discrimination and trust in doctors, pointing out that it could directly affect the physical health of patients who experience discrimination.

While previous research has gleaned important information on trust in doctors and factors that may influence trust, such as discrimination, these factors have not been explored in a national, community-based sample. Because a majority of previous studies examining trust in doctors were completed in clinical samples^2,4,17,19,20^ and many included numerous forms of discrimination, examining discrimination specifically in a medical care setting, as it relates to trust in doctors in a national sample, would improve our understanding of trust among regionally and medically diverse groups of people.

### Present Study

We tested the association between general trust in doctors and age, race, sex, education, urban/rural residence, and previous experience(s) of race-based discrimination in a medical setting in a large cohort study of community-dwelling Black/African American and White adults in the USA. We hypothesized that previous experiences of discrimination in a medical care setting would negatively predict general trust in doctors and would also explain any association between race and trust in doctors.

## METHODS

### Participants and Procedures

The present study is a secondary analysis of REasons for Geographic and Racial Differences in Stroke (REGARDS) study de-identified data that received executive committee approval prior to data analysis. REGARDS is a longitudinal population-based cohort study designed to investigate factors associated with the excess stroke mortality observed among Black/African Americans and residents of the Southeastern USA. At baseline, REGARDS enrolled 30,239 non-Hispanic Black/African American and White adults aged 45 and older between 2003 and 2007.^21^ The community-based sample was recruited by mail and telephone from a nationwide list, and 30% of participants were targeted to be from the stroke belt (a region with a higher mortality from stroke relative to the rest of the nation including Alabama, Mississippi, Louisiana, Arkansas, Tennessee, Georgia, North Carolina, and South Carolina), 20% from the stroke buckle (region within the belt with the most concentrated incidence of stroke along coastal Georgia, North Carolina, and South Carolina), and the remaining 50% outside of these regions. The final baseline cohort included 42% Black/African American adults, 55% women, with 21% from the stroke buckle, 35% from the rest of the stroke belt area, and 44% from the other 40 contiguous states.

REGARDS inclusion criteria were not receiving active treatment for cancer, no medical obstacles that would prevent ongoing participation, no cognitive impairment, not living in or actively attempting to live in a nursing home, and the ability to communicate in English.^21^ REGARDS collected the first wave of data using a computer-assisted telephone interview (CATI). Verbal informed consent was followed by an in-home visit that included physical measures and self-administered questionnaires returned by mail. From 2014 to 2016 (an average of 9 years since participants joined the study), participants completed a second CATI and in-home assessment, including self-administered questionnaires. The institutional review boards of the participating institutions that had access to participant identifying information approved the parent study, and participants provided written informed consent at both the first and second in-home visits. Further details of methods are available elsewhere.^21^

#### Analytic Sample

Our analytic sample included participants who had complete data for all variables of interest and indicated that they had a regular place to go to receive medical care. Neither discrimination in a medical care setting nor general trust in doctors was collected at REGARDS baseline, restricting our sample to those who completed the second in-home visit. Demographic factors of self-reported race, sex, and education were collected at baseline. Age and urban/rural residence were collected at the second in-home visit.

### Measures

#### Trust in Doctors

Participants completed an 11-item measure of general trust in doctors, a modified version of the “Wake Forest Trust in Physicians Scale” referred to as the “General Trust in Doctors Scale” developed by Hall et al.^15,22^ The modification reflects an individual’s general trust in doctors (notably separate from trust in a specific doctor) at the time the measure was completed. This measure was included in the packet of self-administered questionnaires left behind for participants to complete after the second in-home visit.

Participants were instructed to reflect on relationships they have with their doctors and/or the health professionals they see most often for medical care when completing the questionnaire. Examples of statements from this battery include: “Doctors always use their very best skill and effort on behalf of their patients” and “You completely trust doctors’ decisions about which medical treatments are best.” Participants rated their agreement with each of the 11 statements by indicating “5-Strongly Agree, 4-Agree, 3-Neither Agree or Disagree, 2-Disagree, or 1-Strongly Disagree.” Two statements that indicated negative beliefs about doctors were reverse coded so that higher scores indicated greater trust in doctors. Consistent with prior use of the General Trust in Doctors Scale,^15^ we utilized a sum score of the trust items (range 11–55) and restricted the sample to participants who had regular access to medical care. Having regular access to medical care was indicated by answering the question, “Is there a place that you usually go to when you are sick or need advice about your health?” with response options for “No, Yes, Yes—more than one, or don’t know.”

#### Discrimination

Participants completed Krieger et al.’s nine-item “Experiences of Discrimination” (EOD) measure examining lifetime experiences of discrimination.^23^ This measure included an item isolating experiences of discrimination when “getting medical care” at any point over their lifetime based on race, ethnicity, or skin color. Their answers were measured on a 4-point scale, “Have not experienced, one time, two or three times, or four or more times.” We dichotomized the experience of discrimination in a medical care setting based on the highly skewed distribution of participant responses (93% had not experienced; 3% one time; 2% two or three times; 1% four or more times).

#### Demographic Variables

Demographic variables of age, race, sex, level of education, and urban/rural setting were included in regression models. Age was used as a continuous variable. Race and sex were categorical variables. Education was measured as an ordinal variable.

Rural-urban commuting area (RUCA) codes^24^ were matched to the census tract where the participant lived at follow-up. As done in previous REGARDS studies,^25,26^ RUCA classifications were separated into three categories: urban (population greater than or equal to 50,000), large city/town (hereafter referred to as mixed; population between 10,000 and 49,999), or small rural (hereafter referred to as rural; population less than or equal to 9999).

### Analytic Strategy

First, bivariate correlations were examined between measures of interest. Next, we examined the relationship between demographic factors and general trust in doctors in a linear regression model. In a second model, we added previous discrimination in a medical setting as a predictor to examine whether discrimination explained any associations between demographic factors and trust in doctors.

To explore associations between discrimination and race, we conducted a formal mediation analysis using the *medeff*package in Stata.^27^ This package allowed us to test the treatment effect of race on trust in doctors via experiences of medical care discrimination. The *medeff*package is used to test mediation and has the flexibility to incorporate a binary mediator variable via a logistic regression model and produces estimates for the direct effect and total effect (average treatment effect).^20^

## RESULTS

**Figure 1:**
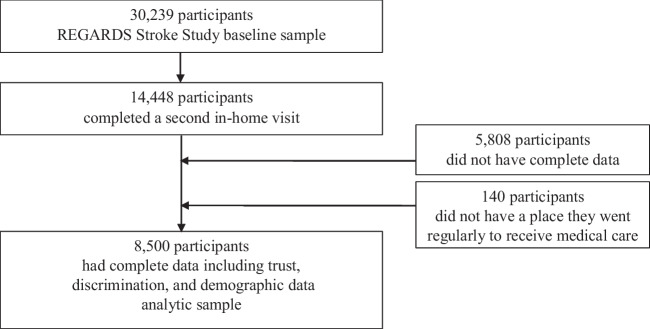
Sample flowchart

The derived analytic sample was *n* = 8500 (Fig. 1). The average age of our sample was 73 years (Table 1). About 31% identified as Black/African American, and 56% identified as female. The average level of education was “some college.” Approximately 75% of the participants lived in an urban area, 12% lived in a mixed urban/rural setting, and 13% lived in a rural area. While about 7% of the overall sample reported experiencing discrimination in a medical care setting, 17% of Black/African American participants reported experiencing discrimination in a medical care setting compared to 2% of White participants. The average score on the General Trust in Doctors Scale was 39, somewhere between neutral and slightly positive.

**Table 1:** Sample Characteristics

Characteristic (*n* = 8500)	*M* (SD)	*n* (%)
Age (years) at follow-up	72.5 (8.4)	
Race (Black/African American)		2640 (31)
Sex (female)		4791 (56)
Education (1–4)		
(1) Less than high school		556 (7)
(2) High school graduate		1938 (23)
(3) Some college		2208 (26)
(4) College graduate and above		3798 (45)
Residence		
Urban		6370 (75)
Mixed		1044 (12)
Rural		1086 (13)
Discrimination (yes)		554 (7)
General trust in doctors (11–55)	38.8 (7.7)	

Discrimination: reflects whether participants had experienced discrimination when receiving medical care; general trust in doctors: lower scores reflect less trust in doctors

Bivariate correlations between all variables ranged from small to medium strength (using Pearson’s R guidelines; Table 2). Discrimination was positively correlated with identifying as Black/African American or female and negatively correlated with age, education, and trust. Trust in doctors was positively correlated with age and negatively correlated with being Black/African American, having higher education, and being female.

**Table 2:** Correlates of Trust in Doctors

Variables	Age	Race	Sex	Education	Rural	Discrimination	Trust
Age	1.00	.	.	.	.	.	.
Race	−0.08*	1.00	.	.	.	.	.
Sex	−0.09*	0.15*	1.00	.	.	.	.
Education	−0.08*	−0.19*	−0.10*	1.00	.	.	.
Rural	−0.03*	−0.17*	0.00	−0.07*	1.00	.	.
Discrimination	−0.07*	0.30*	0.06*	−0.05*	−0.04*	1.00	.
Trust	0.12*	−0.03*	−0.10*	−0.05*	0.01	−0.14*	1.00

Race: 1 = Black/African American participants, 0 = White participants; sex: 1 = female, 0 = male; rural: 1 = population less than 9999

^*^p < 0.05

The first regression model (Table 3) showed that being female (*b* = −1.41, *p* < 0.05), identifying as Black/African American (*b* = −0.40, *p* < 0.05), and having a higher level of education (*b* = −0.45, *p* < 0.05) were each independently associated with lower trust in doctors, while older age (*b* = 0.10, *p* < 0.05) was associated with higher trust in doctors. Urban/rural population size was not significantly associated with trust in doctors.

**Table 3:** Covariate-Adjusted Associations with Trust in Doctors

Variables	Demographic model	Discrimination model
	Estimate^a^ (SE), *β*	Estimate (SE), *β*
Age	0.10 (0.01), 0.10*	0.09 (0.01), 0.10*
Black/African American	−0.40 (0.19), −0.02*	0.28 (0.20), 0.02
Female	−1.41 (0.17), −0.09*	−1.39 (0.17), −0.09*
Education	−0.45 (0.09), −0.06*	−0.44 (0.09), −0.06*
Mixed urban/rural residence	−0.23 (0.26), −0.01	−0.20 (0.25), −0.01
Rural residence	0.10 (0.25), 0.00	0.14 (0.25), 0.01
Discrimination		−4.27 (0.35), −0.14*

*SE*, standard error of *b*; *β*, standardized estimates

Race: 1 = Black/African American participants, 0 = White participants; sex: 1 = female, 0 = male; mixed urban/rural residence: 1 = population between 10,000 and 49,999; rural residence: 1 = population less than 9999; reference group urban = population greater than or equal to 50,000

^a^Estimates represent *b*

^*^p < 0.05

In the second regression model including discrimination in a medical care setting (Table 3), the relationship between race and trust in doctors was completely attenuated (*b* = 0.29, *p* = 0.155). Discrimination had a negative association with trust in doctors (*b* = −4.27, *p* < 0.05), indicating around a 4-point reduction in trust compared to participants who had not experienced discrimination in a medical care setting. Estimates were essentially unchanged after adjustment for discrimination in a medical setting for associations between trust in doctors and age (*b* = 0.09, *p* < 0.05), female sex (*b* = −1.39, *p* < 0.05), and education (*b* = −0.44, *p* < 0.05).

### Mediation Analysis

**Figure 2:**
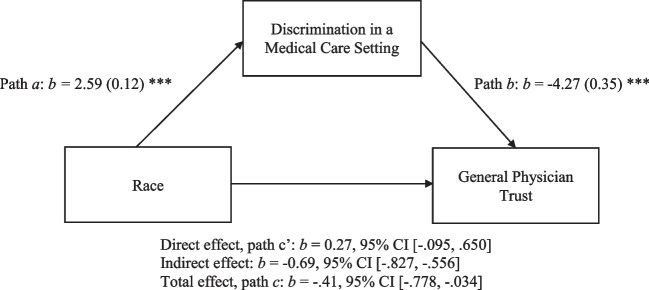
Mediation analysis

There was a significant total association between race and general trust in doctors (*b* = −0.41, 95% CI[−0.778, −0.034]). The association between race and discrimination was positive and statistically significant (path *a*: *b* = 2.60, *p* < 0.001) and the relationship between discrimination and trust in doctors was negative and statistically significant (path *b*: *b* = −4.27, *p* < 0.001). After accounting for discrimination in the model, the direct effect between race and trust in doctors was not significant (*b* = 0.27, 95% CI[−0.095, 0.650]) but the indirect effect was statistically significant (*b* = −0.69, 95% CI[−0.827, −0.556]; see Fig. 2).

## DISCUSSION

We found that identifying as Black/African American, female, and having more education each predicted less general trust in doctors, while older age predicted more trust. Urban/rural residence was not significantly associated with trust. Discrimination predicted less general trust in doctors and mediated the relationship between being Black/African American and lower general trust in doctors. Estimates for associations between other demographic variables and trust remained essentially unchanged after adjustment for discrimination.

Regarding demographic factors, our finding that older age was related to greater trust in doctors is supported by previous research on older adults’ institutional trust in the healthcare system and physicians.^10,28^ It is also important to consider this finding within the context of generational cohorts wherein each group of same-age peers has lived through different historical contexts, such as the increase in education levels over time,^29^ that may contribute to their view of doctors. Regarding sex, we found that identifying as female was significantly positively correlated with experiences of discrimination in a medical care setting and was related to lower trust in doctors. Notably, this association was not found in the original research on the development and validation of the General Trust in Doctors Scale using a smaller sample of 502 adults (339 females, 163 males),^15^ nor was it found in a sample of 563 older adults (348 females, 215 males) in a rural setting.^10^ Interestingly, higher education was related to less trust in doctors, another novel finding compared to a study of older adults in a rural setting in which 36% of the sample had less than a high school education (versus 7% in our sample).^10^ It is possible that those with higher education would have a greater foundation of knowledge and hence greater confidence to critique their healthcare providers, but more research is needed in this area. This education-trust association is in line with our finding that older participants had more trust in doctors than younger participants, as younger age was correlated with more education in our sample, a trend reflected in the general population.^29^

Consistent with some previous literature,^11,30^ we found that Black/African American adults had lower trust in doctors. This relationship between race and trust in doctors, specifically in the USA, has a long history based on racial injustice in the healthcare system and on medical research, a prominent example being the United States Public Health Service Untreated Syphilis Study.^31^ However, our findings suggest that personal experiences of discrimination, which are modifiable, contribute to patients’ lower trust in physicians currently.

While it may not be surprising that previous experience of discrimination in a medical care setting was associated with lower trust in doctors, it is notable that discrimination fully mediated the relationship between race and lower trust in doctors, indicating discrimination as a potential underlying mechanism. This may also help to explain mixed findings for the relationship between race and trust in previous studies^10,12^ and supports the idea that the negative relationship observed between race and trust in doctors is not “simply” due to race but is based on personal discriminatory experiences in the healthcare system faced more often by Black/African American individuals. Prior research supports similar conclusions, linking experiences of discrimination with overall medical mistrust and perceived racism in healthcare.^32^

Race and experiences of discrimination were strongly correlated in our sample, meaning that Black/African American participants faced more previous experiences of discrimination. It is important to continue identifying barriers to trust that may exist between patients and doctors to improve healthcare administered to patients, especially in Black/African American individuals who have historically faced greater harm from medical research and healthcare policy^33^ and continue to experience ongoing interpersonal racism.

Minimizing and eliminating personal experiences of discrimination in medical settings can take many possible avenues, as evidenced by ongoing research in antiracism.^34^ For instance, increased education and healthcare team training in cultural humility may help to avoid experiences of discrimination. Practicing cultural humility (that is, acknowledging one’s own biases and limitations out of respect for the patient’s cultural background) is important to maintain flexibility in the provision of patient care by placing a greater emphasis on patient goals and beliefs.^35^

In this vein, important takeaways from qualitative work concerning the experiences of Black/African American adults with healthcare providers include a desire for a more person-centered approach from physicians, not assuming patient preferences based on cultural stereotypes, and more thorough explanations of treatment plans.^36^ In addition, this work has emphasized addressing discrimination via provider education about microaggressions, improved channels for patients to report instances of discrimination, and empowering the patient via improved communication and increased decision-making about their care.^34^ These suggestions that promote more culturally appropriate practices at the provider level would help to eliminate personal experiences of discrimination.

### Limitations

Because our analyses were cross-sectional, causal inferences cannot be determined. However, the primary measures of interest were naturally lagged, as lifetime experience(s) of medical care discrimination had to occur sometime before data collection, while general trust in doctors reflected participants’ beliefs during the moment that they completed the questionnaire. In addition, a vast majority of our sample had health insurance (95% insured at baseline and 99% at follow-up). Therefore, despite our use of a national community-based sample, these results may not be generalizable to the uninsured population or people without a regular location to receive medical care. In addition, only one item from the EOD measure was used, although it has only been validated in its full and adapted forms.^23^

### Future Directions

Exploring methods for the repair of relationships after discrimination has occurred is an important next step in future work. Given that experiences of discrimination in this study could have occurred at any point over the lifetime in our sample of middle-aged and older adults and were still significantly associated with current trust in doctors, it is possible that experiences of discrimination could influence trust in doctors for years to come. Therefore, researching the effect of intentional work to repair relationships between doctors and individuals who have experienced discrimination is needed. In addition, further research on the efficacy of structural improvements in healthcare is needed. Recent research suggests that efforts toward reducing discrimination in healthcare settings at the structural level could include increasing the compositional diversity of healthcare providers, such as doctors.^37^

### Conclusions

Our findings highlight (1) the occurrence of more discrimination in medical settings experienced by Black/African American adults in our sample, (2) the existence of reduced trust in doctors among Black/African American adults in our sample, (3) the link between personal experiences of discrimination in medical care settings and reduced trust in doctors, and (4) the key role of personal experiences of discrimination in linking race to reduced trust in doctors. These key takeaways point to the need for ongoing efforts toward eliminating discriminatory care practices that have effects that do not end in the doctor’s office, but ripple out to impact individual health behaviors.^3,4,9^

## Data Availability

Requests related to REGARDS data access should be sent to regardsadmin@uab.edu. More information can be found here: https://www.uab.edu/soph/regardsstudy/researchers.
